# BioRxToolbox: a computational framework to streamline genetic circuit design in molecular data communications

**DOI:** 10.1093/synbio/ysae015

**Published:** 2024-11-07

**Authors:** Merve Gorkem Durmaz, Neval Tulluk, Recep Deniz Aksoy, Huseyin Birkan Yilmaz, Bill Yang, Anil Wipat, Ali Emre Pusane, Göksel Mısırlı, Tuna Tugcu

**Affiliations:** Department of Computer Engineering, NETLAB, Bogazici University, Bebek, Istanbul 34342, Turkiye; Department of Computer Engineering, NETLAB, Bogazici University, Bebek, Istanbul 34342, Turkiye; Department of Computer Engineering, NETLAB, Bogazici University, Bebek, Istanbul 34342, Turkiye; Department of Computer Engineering, NETLAB, Bogazici University, Bebek, Istanbul 34342, Turkiye; School of Computing, Newcastle University, Newcastle upon Tyne NE4 5TG, United Kingdom; School of Computing, Newcastle University, Newcastle upon Tyne NE4 5TG, United Kingdom; Department of Electrical and Electronics Engineering, Bogazici University, Bebek, Istanbul 34342, Turkiye; School of Computer Science and Mathematics, Keele University, Keele, Staffordshire ST5 5BG, United Kingdom; Department of Computer Engineering, NETLAB, Bogazici University, Bebek, Istanbul 34342, Turkiye

**Keywords:** molecular communications, genetic circuits, model-driven design, receiver design, intersymbol interference

## Abstract

Developments in bioengineering and nanotechnology have ignited the research on biological and molecular communication systems. Despite potential benefits, engineering communication systems to carry data signals using biological messenger molecules and engineered cells is challenging. Diffusing molecules may fall behind their schedule to arrive at the receiver, interfering with symbols of subsequent time slots and distorting the signal. Existing theoretical molecular communication models often focus solely on the characteristics of a communication channel and fail to provide an end-to-end system response since they assume a simple thresholding process for a receiver cell and overlook how the receiver can detect the incoming distorted molecular signal. In this paper, we present a model-based and computational framework called BioRxToolbox for designing diffusion-based and end-to-end molecular communication systems coupled with synthetic genetic circuits. We describe a novel framework to encode information as a sequence of bits, each transmitted from the sender as a burst of molecules, control cellular behavior at the receiver, and minimize cellular signal interference by employing equalization techniques from communication theory. This approach allows the encoding and decoding of data bits efficiently using two different types of molecules that act as the data carrier and the antagonist to cancel out the heavy tail of the former. Here, BioRxToolbox is demonstrated using a biological design and computational simulations for various communication scenarios. This toolbox facilitates automating the choice of communication parameters and identifying the best communication scenarios that can produce efficient cellular signals.

## Graphical Abstract

**Figure:**
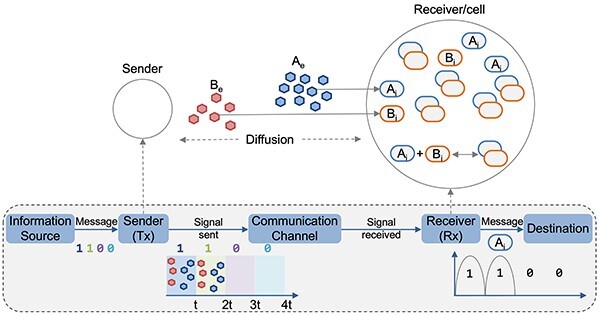

## Introduction

Cells communicate with the environment and each other to maintain life [1]. Examples include single-cell organisms, such as bacteria, that are organized in microsocieties. Naturally, signaling molecules are used as information carriers. Understanding and controlling the mechanisms between sender and receiver cells are essential for communications engineers to design novel applications. Synthetic genetic circuits [2, 3] can offer a rewarding tool for molecular communication systems to go beyond the diffusion of signaling molecules to carry and modulate information between sender and receiver cells. However, the application of integrative approaches that consider both intracellular and intercellular dynamics of signaling molecules to develop robust and biological communication systems, minimizing noise and signal distortion, is limited.

Incorporating the dynamics of underlying transmission channels has several advantages in developing nano- and micro-scale biological communication systems. Inspired by nature, molecular communication systems are bio-compatible [4]. The transmitted molecules propagate freely, and molecular communication via diffusion (MCvD) has no external energy requirements [5].

**Figure 1.**
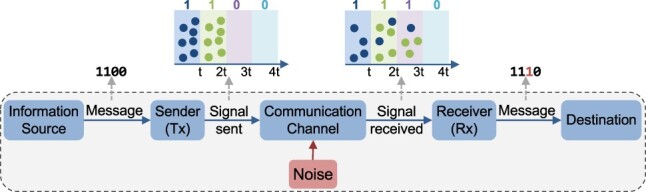
The dashed box shows Shannon and Weaver’s model of communication. Here, this model is adapted for MCvD. The message encoded as a signal by the sender is sent through a communication channel where noise may affect the signal before it is delivered. As demonstrated, the example 1100 message with four symbols is incorrectly decoded as 1110 by the receiver due to molecules arriving late in subsequent slots and choosing a short symbol duration. The situation is known as ISI.

A well-known model of a communication system developed by Shannon and Weaver [6] consists of five key elements: an information source that generates the message, a sender or transmitter (*Tx*) that encodes the message into a communication signal, a communication channel in which the signal propagates, a receiver (*Rx*) that decodes or translates the received signal back to information, and a destination node that processes the incoming information (Fig. 1). A message in an MCvD context can be considered as a sequence of bit-0 and bit-1 symbols. Generally, communication is carried out in a time-slotted manner, and the duration for the transmission of a single symbol is called symbol duration. A bit-1 symbol can be encoded using a group of signaling molecules that are released from a sender for a given duration and propagate through the communication channel, and a bit-0 symbol represents the state when no molecules are released.

Developing communication channels and encoding information can be affected by inherent noise and interference due to other molecules in the intercellular environment and the dispersion of the molecules over time during diffusion [7]. Noise is undesired and can be defined as any interference that changes the received signal, typically in a destructive way [6]. Diffusing molecules may not arrive at a receiver cell on their predestined time slots and may interfere with the subsequent bit transmissions [8]. As a result, when molecules are released from a sender to encode a particular symbol, the receiver may decode this symbol incorrectly (Fig. 1). This situation causes significant intersymbol interference (ISI), which is considered one of the major challenges in diffusion-based communication systems that hinder communication [9, 10].

Engineering an MCvD system and ISI mitigation can become even more challenging when living cells act as receivers. Signaling molecules moving in the intercellular medium can trigger adverse cellular responses, propagating and amplifying the inherent noise [11]. Moreover, due to different timescales in intercellular diffusion dynamics and intracellular biochemical reactions, received signals may further interfere with the subsequent cellular signals. Although these issues have been studied in the molecular communications literature, extending or implementing proposed mechanisms inside the cell remains an ongoing challenge. One way to facilitate the design of predictable systems is to apply model-based design approaches, which may involve integrating multi-scale mathematical models [12, 13] to simulate the dynamics of cellular response to messenger molecules that carry information for signaling and diffuse across a communication channel.

Different theoretical approaches have been proposed to handle ISI [14–16]. However, these approaches do not address the issue of how diffusing molecules are converted to intracellular signals that affect a system’s response and noise when genetic receivers are employed. For example, Noel *et al*. [17] proposed adding enzymes into the propagation channel. Enzymes degrade information molecules and prevent stray molecules from interfering with future transmissions. However, the signal’s intensity may also be reduced, and the cellular response is not controlled. Tepekule *et al*. [18] proposed a molecular transition shift keying technique in which the presence of two different types of molecules (Type A and Type B) is used to encode a bit-1 symbol, and the absence of these molecules represents a bit-0 symbol. The choice of molecule type to encode bit-1 depends on the following bit-0 or bit-1 symbol. If the next symbol is bit-0, Type B molecules are sent. If the next symbol is bit-1, Type A molecules are sent. This strategy ensures that only Type B molecules are released before bit-0, and the accumulation of molecules and ISI are restrained [18]. Another proposed solution is the pre-equalization method [19], which involves transmitting two different molecule types from a sender: Type A information encoding molecules and Type B destructive molecules. In this approach, Type B molecules eliminate the effect of the stray Type A molecules. The impact of the destructive molecule is imitated by employing a subtraction operation at the receiver. However, this theoretical approach also does not address minimizing ISI at the cellular level.

Different modulation techniques have also been proposed to encode information using molecular communication systems. These techniques involve controlling the concentration of the transmitted molecules from the sender [20], the type of the transmitted molecules [21], and the release time of the transmitted molecules within a communication time slot [22].

The movement of transmitted molecules in a molecular communication channel can be modeled by the diffusion process or the Brownian motion. In a fluidic environment without any flow, molecules move randomly [23]. When diffusing molecules reach receiver cells, they may activate some processes or yield information bits after a demodulation process. Therefore, evaluating the expected number of received molecules is critical for designing an effective MCvD system that involves receiver cells. Moreover, the selection of genetic parts and their interactions may affect the activation of a synthetic genetic circuit inside a receiver. It is desirable to incorporate models of genetic circuits to design efficient molecular communication systems and understand the effect of ISI on cellular response.

Various modeling formalisms exist to analyze the dynamic behavior of genetic circuits. For example, the Systems Biology Markup Language (SBML) [24] standardizes the representation of biochemical reactions. Tools such as the Complex Pathway Simulator (COPASI) [25] can simulate SBML models to gain insight into emerging cellular behavior. Moreover, standardization efforts are essential to exchange information between tools without data loss. Synthetic genetic regulatory circuits can be computationally represented using the Synthetic Biology Open Language (SBOL) [26, 27]. SBOL designs can include constraints to capture the sensing of external molecules and descriptions of intended biochemical reactions. This qualitative information can consequently be used to create quantitative models that can be simulated.

SBML and SBOL are increasingly used for the model-driven design of synthetic genetic circuits. Moreover, the Virtual Parts Repository (VPR) [28, 29] converts annotated SBOL documents to SBML models to automate the generation of computational models of genetic circuits [29, 30]. VPR provides reusable, modular, and mathematical models of biological components such as promoters, ribosome binding sites, and coding sequences. These models can be connected to create hierarchical and simulatable models of desired systems. This model-driven approach is ideal for designing and optimizing genetic circuits and computationally exploring large design spaces of biological systems.

Here, we present a computational modeling approach to facilitate the design of molecular communication systems that can be coupled with engineered cells to encode and send information using biological molecules. This approach is workflow based and integrates the modeling efforts in molecular communications and synthetic biology. Our modeling framework, called BioRxToolbox, can aid in producing efficient data signals, allowing computationally optimizing key communication parameters (such as symbol duration and the number of molecules released) via design space exploration and computational simulations. Furthermore, the Period Finder algorithm presented in this paper minimizes signal interference by extending the pre-equalization method [19] to address the effects of intercellular and intracellular signaling processes.

## Materials and methods

BioRxToolbox was implemented in MATLAB and Java. Diffusion and cellular models were integrated in a multi-scale approach, and the evaluation of different communication scenarios was automated via simulations. The cellular response to diffusing signaling molecules was controlled via a genetic circuit. Diffusion parameters and the initial model of the circuit were used as parameters, and the resulting models were customized for each scenario.

### Diffusion modeling

According to Brownian motion, the movement of particles in a three-dimensional space can be represented via three independent displacements, one for each dimension, where each displacement follows a normal distribution with zero mean and *σ*^2^ variance, denoted as follows:


(1) $$\Delta x,\,\Delta y,\,\Delta z\, \sim \,{\mathcal N}\,(0,\,{\sigma^2})$$

where $\sigma = \sqrt {2D\Delta t} $, *t* is the time, and *D* is the diffusion coefficient that describes the mobility of molecules [23].

Assuming a simple MCvD channel without flow, the expected fraction of diffusing Type A molecules (*A_e_*), which will reach and be absorbed by an *Rx* receiver during the time frame *t_k_*, can be calculated as follows:


(2) $$\,\,\,\,\,\,\,\,\,\,\,\,\,\,\,\,\,\,\,\,\,\,\,\,\,\,\,\,\,\,\,\,\,\,\,\,\,\,\,\,\,\,\,\,\,\,\,\,\,\,\,\,E\left[ {N_{{A_e}}^{Rx}\left( {{t_k}} \right)} \right] = \,N_{{A_e}}^{Tx}\left\{ {{F^{Rx}}\left( {t_k^ + } \right) - {F^{Rx}}\left( {t_k^ - } \right)\,} \right\}$$

where $E\left[ . \right]$ is the expectation operator, $N_{{A_e}}^{Tx}$ is the number of emitted molecules, ${F^{Rx}}\left( t \right)$ is the time-dependent formula for the expected cumulative fraction of arriving molecules [31], $t_k^ - \,$ is the start, and $t_k^ + $ is the end of time frame *t_k_* [18]. For a simple and symmetric topology like a point transmitter and a single spherical absorber, ${F^{Rx}}\left( t \right)$ is known analytically [31].

**Figure 2.**
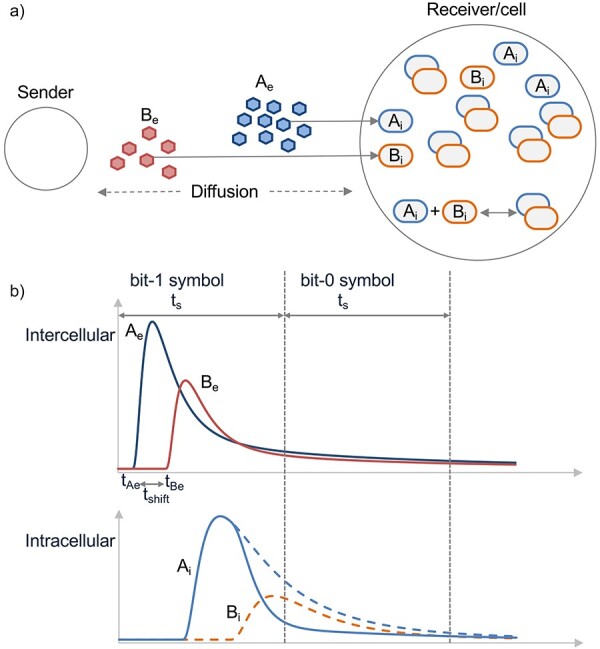
(a) The schematic representation of the general framework for BioRxToolbox, where intercellular *A_e_* and *B_e_* signals are converted to intracellular *A_i_* and *B_i_* signals. (b) This is a hypothetical illustration where bit-0 represents no transmission and bit-1 represents the transmission of *A_e_* and *B_e_* molecules. In the upper graph, *t_Ae_* is the transmission time of *A_e_, t_Be_* is the transmission time of *B_e_, t_Be_ *− *t_Ae_* is the *t_shift_* delay, and *t_s_* denotes the symbol duration, the time slot that the receiver can detect a bit-1 or bit-0 symbol. *A_e_* is converted to *A_i_* with a delay due to diffusion and cellular processes. *B_i_* molecules mitigate the heavy tail of *A_i_* by sequestering *A_i_* molecules. The remaining *A_i_* signal denotes the result of the *A_i_* − *B_i_* biological subtraction operation.


In the general model shown in Fig. 2, the resulting proteins inside *Rx* (*A_i_* and *B_i_*) can bind together. Therefore, *B_i_* can eliminate the effect of stray molecules at the receiver. If *A_i_* exceeds a certain concentration level (*λ*) in time slot *t_k_, Rx* interprets the received symbol as bit-1, and bit-0 otherwise. This process can be represented as follows:

$$S\left[ {{t_k}} \right] = \left\{ {\begin{array}{*{20}{c}}{bit - 1\,\,\,\,N_{{A_i}}^{Rx}\left[ {{t_k}} \right] \ge \lambda }\\\,\\{bit - 0\,\,\,N_{{A_i}}^{Rx}\left[ {{t_k}} \right] < \lambda }\end{array}} \right.$$

where *S*[*t_k_*] is the received or decoded symbol in the time slot *t_k_*.

### Modeling the cellular behavior

The genetic circuit design builds upon gene regulatory networks involving transcriptional and translational processes. Molecular interactions between different circuit components convert the intercellular signals into corresponding cellular signals to control cellular response. VPR2 [29] was used to create models of biological parts and interactions and connect them in order to create simulatable SBML [32] models. Automating the model construction process was facilitated by the SVPWrite language [29] to specify the order and types of biological parts. For example, the “prom1:prom;rbs1:rbs;cds1:cds;ter1:ter” input specifies a single transcriptional unit where “prom1” is a promoter, “rbs1” is an RBS; “cds1” is a CDS, and “ter1” is a terminator. The SVPWrite descriptions were then converted into an SBOL document [26], which was extended with information about molecular interactions and annotated with parameters. Hierarchical system models were derived via VPR2’s SBOL-to-SBML conversion. Diffusion dynamics were integrated via molecular communication parameters for design space exploration, and customized SBML events were added for input signals using the JSBML [33] Java library to analyze and evaluate cellular dynamics for each communication scenario. The simulation of resulting SBML models was automated using the COPASI Java bindings [25].

### Evaluating communication scenarios

Parameters, such as the number of molecules released by the sender and the delay between input signals, were used to derive custom genetic circuit models, each representing a possible communication scenario. The molecular eye (MOL-eye) [34] performance metric was adopted to evaluate these scenarios. MOL-eye is similar to the “eye” diagram that is used for measuring the quality of signals in conventional communication schemes and is adapted to molecular communications.

## Results

The computational modeling approach presented here was developed to design communication systems using molecular and biological communication channels. This process involves coupling intracellular and extracellular processes with diffusion dynamics and three-dimensional molecular channel propagation. Hence, BioRxToolbox facilitates designing biologically plausible and diffusion-based cellular reception processes, which are generally overlooked in the molecular communications research community. The information is encoded as sequential bits, each representing a group of molecules a sender releases. As a result, a response signal is created at the receiver via the accumulation of cellular molecules. The coupling of intercellular and intracellular mechanisms is implemented as a workflow in which an MCvD system with a new pre-equalizer method minimizes cellular ISI. We demonstrate this approach computationally using a receiver design based on synthetic and bacterial genetic regulatory networks to decode information.

### A pre-equalizer for engineered receiver cells

In this work, we also present a cellular pre-equalizer method based on previous work [8, 19]. This new method involves two input signals emitted from the sender and two additional cellular signals inside the receiver (Fig. 2a). The external input signals (*A_e_* and *B_e_*) together carry a single bit of data to reduce ISI. Bit-0 corresponds to no transmission, while bit-1 implies both *A_e_* and *B_e_* molecules being sent over a specific period. The first input signal (Type A) is the data carrier, and the second input signal (Type B) removes the heavy tail of the former (Fig. 2b). *A_e_* and *B_e_* signals are transformed into intracellular *A_i_* and *B_i_* signals. *A_i_* is the observable molecule that relays the signal, and *B_i_* is the antagonist to cancel out the right amount of *A_i_* and mitigate the adverse effects of ISI.

It is crucial to comply with the processing rates of receiver cells when sending sequential data bits. This process requires maintaining a specific level of messenger molecule concentration. Moreover, to eliminate the heavy tail of *A_i_* at the *Rx* receiver, *B_e_* is emitted *t_shift_* seconds after *A_e_* is released from the *Tx* sender.

### Biological use case

Employing a pre-equalizer to minimize interference requires a subtraction operation. Inside the receiver cell, *A_e_* activates the production of *A_i_*, and *B_e_* activates the production of *B_i_*. The difference between *A_i_* and *B_i_* molecules is evaluated using a design pattern involving two molecules that can bind together [35]. *B_i_* sequesters *A_i_* and reduces the number of stray *A_i_* molecules remaining after the symbol duration. The *A_i_* molecules that are not bound represent the result of the subtraction operation.

**Figure 3.**
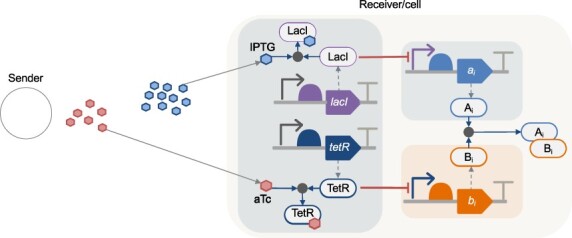
Representation of the system model used in simulations to perform the biological subtraction operation. Remaining *A_i_* molecules over a defined symbol duration period represent a data bit and act as TFs to regulate cellular response. IPTG and aTc are examples of *A_e_* and *B_e_*. The expression of *A_i_* and *B_i_* is activated upon sensing IPTG and aTc.

BioRxToolbox can be parameterized with different *A_e_* and *B_e_* signals and models of genetic circuits that sense these signals. To demonstrate our approach, isopropyl-β-D-1-thiolgalactopyranoside (IPTG) and anhydrotetracycline (aTc) diffusing molecules were selected to represent *A_e_* and *B_e_* signals (Fig. 3). LacI and TetR repressors inhibit the production of *A_i_* and *B_i_*, respectively. Hence, IPTG activates the production of *A_i_* by inhibiting LacI, and aTc activates the production of *B_i_* by inhibiting TetR. Molecules such as ExsD and ExsA that can bind together represent the *A_i_* and *B_i_* molecules [36, 37]. Here, ExsD and ExsA were chosen since they can act as transcription factors (TFs) to control cellular response. Hence, the genetic circuit’s inputs are data bits formed from external *A_e_* and *B_e_* signals, and its outputs are *A_i_* and *B_i_* TFs. With appropriate communication parameters, the deteriorating effects of ISI molecules can be eliminated by considering the *A_i_ *− *B_i_* difference.

### Communication Period Finder algorithm

The Period Finder algorithm within BioRxToolbox implements the proposed cellular pre-equalizer method to efficiently design communication channels. It evaluates the communication performance and optimizes the pre-equalizer’s parameters, including effective *A_e_*/*B_e_* ratios, to minimize the degrading effects of ISI. Initially, Period Finder generates possible signal propagation scenarios and then evaluates these scenarios to ensure that each bit-1 or bit-0 symbol persists for a desired duration. These communication scenarios are then scored using MOL-eye diagrams and ranked to identify the most effective scenarios.

#### Algorithm

**Figure 4.**
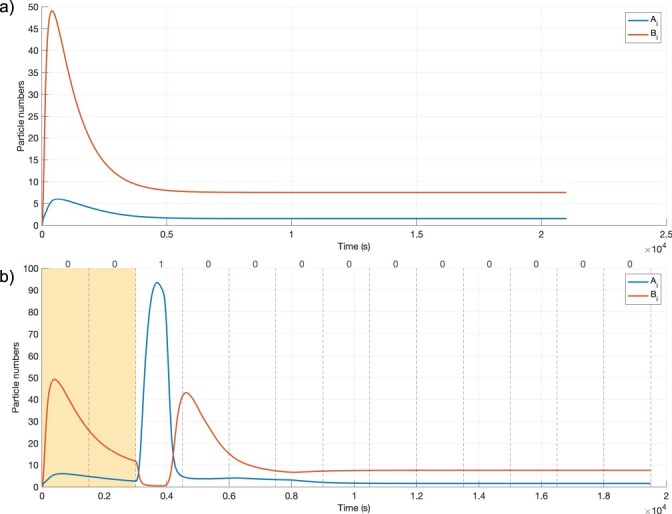
(a) Native state of the *Rx* response *A_i_* (blue lines) and *B_i_* (orange lines) for the 0000000 signal when using a long *t_s_* (3000 s). (b) The *Rx* response for a one-shot signal (0010000000000) for the simulation parameters in Table 1. The *t_shift_* is 900 s to keep the *A_i_* signal large enough (*t_sDefault_* = 1500 s and *α *= 0.15). The first two symbol durations indicate the warm-up period.

Potential communication scenarios are explored according to the total number of molecules (*M*) sent per bit-1 symbol, various *B_e_/M* ratios (*α*), and various delay values between *A_e_* and *B_e_* signals (*t_shift_*) at the sender, together with parameters to optimize the *t_s_* symbol duration (Fig. 2b). The algorithm to find the best communication scenarios is shown in Algorithm 1. BioRxToolbox initially determines the default states of *A_i_* and *B_i_* in a receiver cell without any inputs. After a warm-up period [38], the system reaches an equilibrium state (Fig. 4a). Hence, each simulation is started with two bit-0 symbols corresponding to the warm-up period in order to allow the system to stabilize. It then simulates sending a one-shot signal (Fig. 4b) for various communication scenarios, where a single bit-1 symbol is transmitted to infer optimum *t_s_* values before sending complex data bits. Each simulation corresponds to a communication scenario and involves modeling diffusion, *A_e_* (IPTG) and *B_e_* (aTc) signal construction at the receiver for different bit-1 and bit-0 symbols of the message, and modeling the dynamics of the genetic circuit. Simulation results are saved for further processing, including generating plots and evaluating scenarios according to the MOL-eye performance metric. The algorithm is explained further in the following sections.

**Algorithm 1** Identifying the best communication
 scenarios
1. Obtain the receiver’s native state parameters
 (basal *A_i_* and *B_i_* values) when *A_e_ *= 0 and
 *B_e_* = 0 and calculate the *B_i_*/*A_i_* ratio at the
 end of the simulation.
2. Start all future simulations with the 00 data bits
 to let the system reach the native state before
 sending any bit-1 symbol.
3. Initialize system parameters.
4. *α*: Percentage of *B_e_* molecules as a vector
 (e.g. [0.15,0.6] ∩ 0.05Z for 10 different *α* values
 between 15% and 60%).
5. *t_shift_*: Delay between *A_e_* and *B_e_* as a
 vector (e.g. [0,1000] ∩ 100Z for 11 different
 *t_shift_* values between 0 and 1000 s).
6. For each *α* and *t_shift_* pair, simulate sending
 a one-shot signal (e.g. 0010000000000 data bits).
7. Obtain the *A_i_* and *B_i_* values using an initial
 and unoptimized *t_sDefault_* long enough to observe
 a full bit-1 symbol (e.g. 1500 s).
8. Calculate the optimum *t_s_* when the *B_i_*/*A_i_*
 ratio is close to the native state value after the
 bit-1 symbol is sent.
9. If *t_s_ < t_sMax_*
10. Simulate the system for the intended data bits
 (e.g. 0010111100101) using the *t_s_, α*, and
 *t_shift_* values.
11. Calculate the MOL-eye performance score of the
 communication scenario.
12. Rank the selected communication scenarios using
 the MOL-eye scores.

#### Diffusion-based signal construction

**Figure 5.**
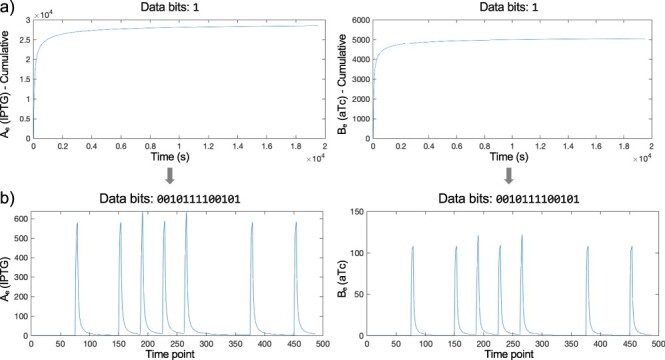
An example signal construction for the 0010111100101 data bits (*α *= 0.15, *t_shift_* = 600 s, *t_sDefault_* = 1500 s). (a) The cumulative time-series data for IPTG (*A_e_*) and aTc (*B_e_*) molecules that arrive at the receiver during a single bit-1 symbol. (b) The derivative IPTG (*A_e_*) and aTc (*B_e_*) signals arriving at the receiver for all bit-0 and bit-1 symbols are constructed with a sampling rate of 40 s. This process is repeated for all six bit-1 symbols of the 0010111100101 data bits.

The cumulative number of diffusing molecules that arrive at a receiver is calculated using the initial quantities of external *A_e_* and *B_e_* released from the sender according to the diffusion parameters in Table 1. For example, Fig. 5a shows the cumulative numbers when *A_e_* is 85% and *B_e_* is 15% (*α *= 0.15) of molecules released. These cumulative data are then used to calculate the derivative values, representing the number of *A_e_* or *B_e_* molecules that reach the receiver during a single bit-1 symbol. This signal construction process is repeated for each bit-1 symbol of the message. For example, Fig. 5b demonstrates the *A_e_* and *B_e_* signals arriving at the receiver for the 0010111100101 data bits. A moving average with a sampling rate of 40 s is used to finalize the results. This process is repeated for each communication scenario, and the results for *A_e_* and *B_e_* are saved in individual CSV files for integration with cellular modeling.

**Table 1.** Simulation parameters and values

Parameter	Value
Diameter of *Rx*	2 µm [39]
Total number of molecules (*M *= *A_e_* + *B_e_*)	3 500 000 [40]
Distance between *Tx* and *Rx*	200 µm [4]
Diffusion coefficients for IPTG (*A_e_*) and aTc (*B_e_*)	600 and 870 µm^2^*/*s [41]
Maximum symbol duration	2000 s

#### Modeling the genetic circuit

BioRxToolbox utilizes the VPR [29] framework to design and model the genetic circuit (Fig. 3). The circuit comprises three devices. The first device senses IPTG (*A_e_*) and aTc (*B_e_*) and produces LacI and TetR proteins. The second device, controlled by LacI, produces *A_i_*, while the third device, controlled by TetR, produces *B_i_*. The devices were initially specified using SVPWrite (Supplementary Tables S1 and S3). An overview of biological interactions represented in each model is summarized in Supplementary Table S2. These interactions include the production of mRNAs and proteins, TF-promoter inhibition, complex formation, binding, unbinding, and degradation. Parameters from an existing toggle switch design [29] and nominal values were used, assuming that each device is deployed using low-copy plasmids (10 copies).

The resulting hierarchical SBML model is customized using different *α, t_shift_, t_s_*, and data bit values for each scenario to integrate diffusion dynamics of molecules released from the sender and arriving at the receiver. BioRxToolbox creates an SBML event for each IPTG and aTc value in CSV files from the signal construction step. These values represent the expected IPTG and aTc molecules at the receiver. BioRxToolbox then automates the simulations to determine *A_i_* and *B_i_* values for evaluations.

#### Optimizing symbol durations

Due to using different amounts of *A_e_* and *B_e_* and the resulting cellular dynamics, receivers require different symbol durations in each scenario to identify data bits correctly without any interference. To infer optimum *t_s_* values, BioRxToolbox initially determines native or basal state parameters with respect to the cellular *B_i_*/*A_i_* ratio using the 0000000 data bits when there are no *A_e_* and *B_e_* present (Fig. 4a).

**Figure 6.**
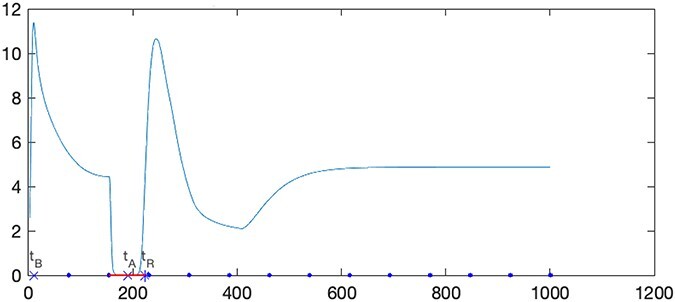
An example of inferring a *t_s_* value (*α *= 0.15, *t_shift_* = 900 s, *t_sDefault_* = 1500 s, and data bits = 0010000000000) for the scenario in Fig. 4b. *A_i_* values are scaled to [0–1000] s to standardize comparisons for different simulation durations. The figure is annotated with *t_B_* (when *B_i_*/*A_i_* is maximum), *t_A_* (when *A_i_* is maximum), and *t_R_* values. The *B_i_/A_i_* ratio approaches the native state’s *B_i_/A_i_ ratio at the t_R_* time point when t>*t_A_* and t>*t_B_*. The inferred and scaled *t_s_* for the bit-1 symbol is shown using the red line, excluding the initial warm-up period for the first two bit-0 symbols. This inferred value is then multiplied by ∆*t* (*t_sDefault_* * length*_bits_*/1000) to calculate the unscaled and optimum *t_s_* value.

BioRxToolbox then infers the optimum *t_s_* values. The system is simulated for each *α* and *t_shift_* pair using a single bit-1 symbol, followed by bit-0 symbols with the 0010000000000 one-shot signal (Fig. 4b). Employing a single bit-1 symbol prevents ISI, and a default *t_s_* (e.g. 1500 s), long enough to observe the bit-1 symbol, is selected. The native state information is used to determine the optimum *t_s_* that takes the system to return to equilibrium, which is the base state of the cell after the bit-1 symbol is received, as demonstrated in Fig. 6. Additional examples are shown in Supplementary Fig. S1.

**Figure 7.**
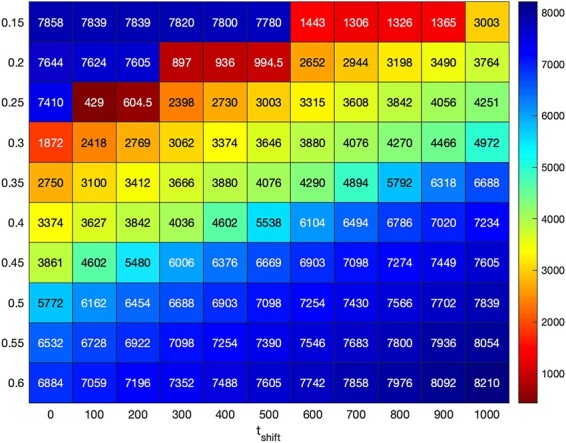
The heatmap shows the optimum *t_s_* values inferred using the 0010000000000 data bits for 110 different communication scenarios corresponding to 10*α* and 11*t_shift_* values. A communication scenario is discarded if *t_s_* *> t_sMax_*. Hence, 10 scenarios represented in red are retained.

The corresponding communication scenario is discarded if the achieved *t_s_* value is greater than the maximum *t_s_* value. Waiting for the equilibrium state to be achieved ensures the system is on hold until the channel is cleared out to send the subsequent symbol, decreasing the effect of ISI. BioRxToolbox reports optimized *t_s_* values using a heatmap (Fig. 7). Out of the 110 scenarios explored, 10 communication scenarios that satisfy the *t_s_* *< t_sMax_* requirement were retained for further evaluations using data communications.

#### Minimizing interference in MCvD

**Figure 8.**
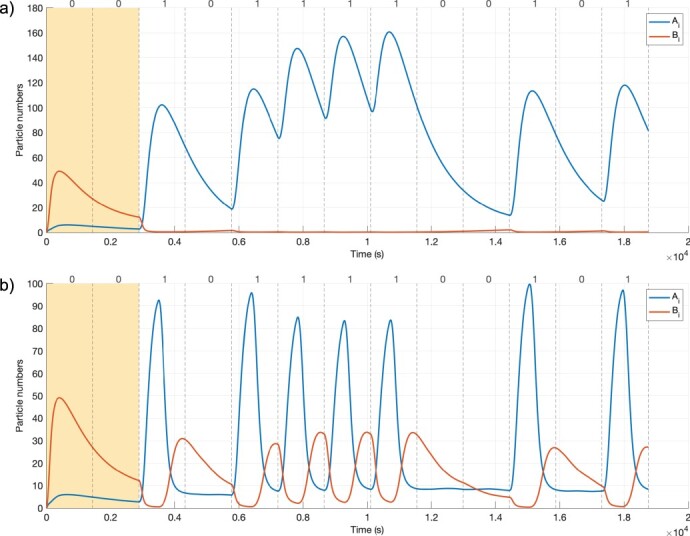
The *Rx* response *A_i_* (blue lines) and *B_i_* (orange lines) of the 0010111100101 signal using the optimum symbol duration inferred (1443 s) without and with the pre-equalizer to minimize ISI. The first two *t_s_* symbol duration slots for the warm-up period are shown in yellow. (a) Response without using the *B_e_* pre-equalizer. After a train of consecutive bit-1 symbols, the signaling molecules accumulate in the channel. The concentration of signaling molecules at the ninth symbol (bit-0) is misleadingly high due to the effect of ISI, which is likely to cause an incorrect detection at the receiver. (b) *Rx* response using the *B_e_* pre-equalizer (*α *= 0.15 and *t_shift_ *= 600 s). As desired, signaling molecules do not start accumulating after the fifth symbol duration. Furthermore, the concentration of signaling molecules at the ninth symbol is as expected since the pre-equalizer mitigates the effect of ISI.

BioRxToolbox simulates the communication scenarios that satisfy the optimum symbol duration criteria using the intended data bits. The simulation results of *A_i_* and *B_i_* for a scenario when the *B_e_* pre-equalizer is not incorporated are shown in Fig. 8a. The bit sequence for this simulation is 0010111100101, and the simulation parameters from Table 1 are applied. The first two bit-0 symbols represent the warm-up period. The absence of the pre-equalizer leads to ISI. After the fifth symbol, consecutive bit-1 symbols result in the accumulation of stray molecules. Consequently, the concentration of *A_i_* data carrier molecules at the ninth symbol (bit-0) is misleadingly higher than expected.

The same communication scenario is shown in Fig. 8b when the *B_e_* pre-equalizer is added to the system. As expected, when the 0010111100101 bit sequence is sent, consecutive bit-1 symbols in the middle no longer result in the accumulation of molecules. Moreover, each bit-1 and bit-0 information is exchanged clearly, demonstrating that the effects of ISI are drastically reduced.

#### Identifying the best communication scenarios

**Figure 9.**
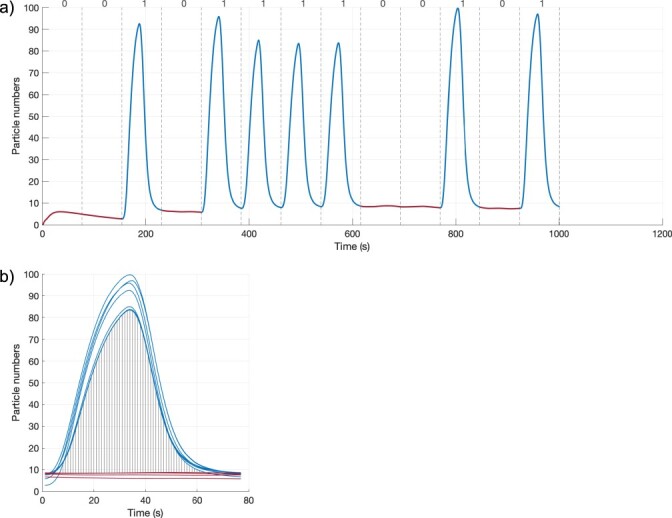
Example demonstrating the MOL-eye performance evaluation for the scenario described in Fig. 8b. Red lines represent *A_i_* when bit-0 symbols are sent, and blue lines represent *A_i_* when bit-1 symbols are sent. (a) *A_i_* values are scaled to [0–1000] s to standardize the evaluation of scenarios. (b) MOL-eye diagram, where successive bit-1 (seven blue lines) and bit-0 (four red lines) values of *A_i_* are superimposed. The first two bit-0 symbols during the warm-up period are not included. The figure shows the opening between the maximum of *A_i_* values during bit-0 symbols and the minimum of *A_i_* values during bit-1 symbols.

BioRxToolbox evaluates the selected communication scenarios by calculating MOL-eye scores and ranking them using the simulation results for the *A_i_* data carrier signal. The results are scaled into the range of 0–1000 s to standardize the performance evaluations (Fig. 9a). An example MOL-eye diagram of a scenario with a high score is shown in Fig. 9b, where the subsequent signals for each bit are superimposed to a single composite graph. The area between the minimum of *A_i_* values during all bit-1 symbols and the maximum of *A_i_* values during all bit-0 symbols is used to evaluate the eye-opening pattern [34]. The differences between the minimum of bit-1 and the maximum of bit-0 values for each time point are added to calculate the score, ignoring negative values. This score is then multiplied by the optimum *t_s_* value. Additional examples are shown in Supplementary Figs. S2 and S3. A scenario is considered better if the corresponding area score is greater. Noise is expected to be the highest when the eye pattern is in the most closed form, making it challenging to distinguish bit-1 and bit-0 symbols. However, the best scenarios identified by the Period Finder algorithm show that the eye-opening is still clear even when the minimum of bit-1 and maximum of bit-0 values are used.

## Discussion

Molecular communication systems offer several advantages in developing biological applications. However, it is challenging to create such applications, which involve encoding and decoding information in environments subject to high noise and external interference. The complexity increases when these communication systems are coupled with cells that act as receivers due to the large number of genetic parts that can be chosen to decode information and control cellular response. Moreover, diffusion-based and cellular processes can be complex and have different timescales. Conducting trial-and-error-based wet-lab experiments can be costly and out of reach for most researchers due to the need for specialized laboratories, equipment, and staff [42].

Simulation environments can provide valuable insights into developing and testing novel communication models. The work presented here involves algorithms, design patterns, and a simulation approach to overcome the obstacles in engineering receiver cells that function via molecular communications and diffusion of molecules to encode and send information.

One of the main challenges in using engineered receiver cells and diffusion-based systems is decoding information due to inherent noise. Our work extends the previously proposed pre-equalizer approach [18] by incorporating two additional cellular signals. A biological subtraction operation for these cellular signals has been defined as a genetic circuit design to improve the molecular channel response, reduce cellular noise, and control cellular response.

BioRxToolbox, using its Period Finder algorithm, can search for successful communication scenarios based on the transmission time differences between the input signaling molecules and their ratios. These scenarios are ranked using the MOL-eye performance metric. Hence, BioRxToolbox can be ideal for automating the exploration of different communication parameters via computational simulations. Other BioRxToolbox parameters, such as the number of total molecules and diffusion parameters, can also be adjusted. For example, Supplementary Fig. S4 shows the variation of symbol durations for different distance parameters. When the distance between the sender and the receiver is increased, fewer molecules reach the receiver due to diffusion. As a result, the number of molecules transmitted may need to be increased, and *B_e_* may need to be released with a higher delay not to fully suppress *A_e_*.

It may be challenging to meet the expectations of generic communication systems while developing biological applications. Here, we explored minimizing ISI by optimizing symbol durations, which can be minutes due to the diffusion of molecules and accumulation of cellular molecules via transcription and translation processes [43]. As a result, a communication scenario involving a series of data bits may take hours.

Another challenge in our experiments is establishing the warm-up period. In a discrete-event system, the system is initially empty and idle. The situation is different in biological systems and can cause inaccurate results due to the leakiness and basal expression of biological molecules [44]. To improve a molecular channel’s efficiency, decoding information in receiver cells should start after a sufficient warm-up period for the system to reach an initial steady state. Hence, the first two symbols are chosen as bit-0 in simulations, and results within the warm-up period are ignored to improve the accuracy of results.

BioRxToolbox, the cellular pre-equalizer, and the genetic circuit design patterns presented here can be utilized in the development of various cellular and molecular communication systems. For example, these concepts can be used in experimental contexts to create and control cellular delivery systems, biological information processors, and biological clocks.

In the future, we plan to extend the BioRxToolbox modeling framework by incorporating stochastic simulations. In this work, deterministic models were used to understand the overall system behavior due to the large number of molecules involved, assuming that the molecules inside the receiver are well stirred [45]. We also did not consider the chemical nature of *A_e_* and *B_e_* molecules in membrane diffusion [46]. In the future, models can be fine-tuned to incorporate such mechanisms.

BioRxToolbox presented here demonstrates how efforts in molecular communications and synthetic biology can be combined to provide an integrated view of intracellular and intercellular processes to design novel communication systems. Our approach allows validating molecular communication designs *in silico* and identifying suitable system parameters computationally to inform wet-lab experiments.

## Supplementary Material

ysae015_Supp

## Data Availability

The open-source BioRxToolbox project, including the source code, genetic circuit designs, and mathematical models, is publicly available via GitHub at https://github.com/dissys/biorxtoolbox.
