# Supplementary material for: BioRxToolbox: a computational framework to streamline genetic circuit design in molecular data communications
Source: Synth Biol (Oxf). 2024 Nov 7;9(1):ysae015. doi: 10.1093/synbio/ysae015 (PMC11636266; doi:10.1093/synbio/ysae015)
Supplement: ysae015_Supp [file ysae015_supp.zip › suppl_data/Supplementary_Material.pdf]

Supplementary Materials

Inferring optimum symbol durations

Optimum symbol durations were inferred for 110 communication scenarios for ten α and 11 t_{shift} values. Figure S1 shows how the optimum symbol durations (t_s) are identified when α is 0.15. A communication scenario is chosen for further evaluation if the inferred symbol duration does not exceed a maximum value.

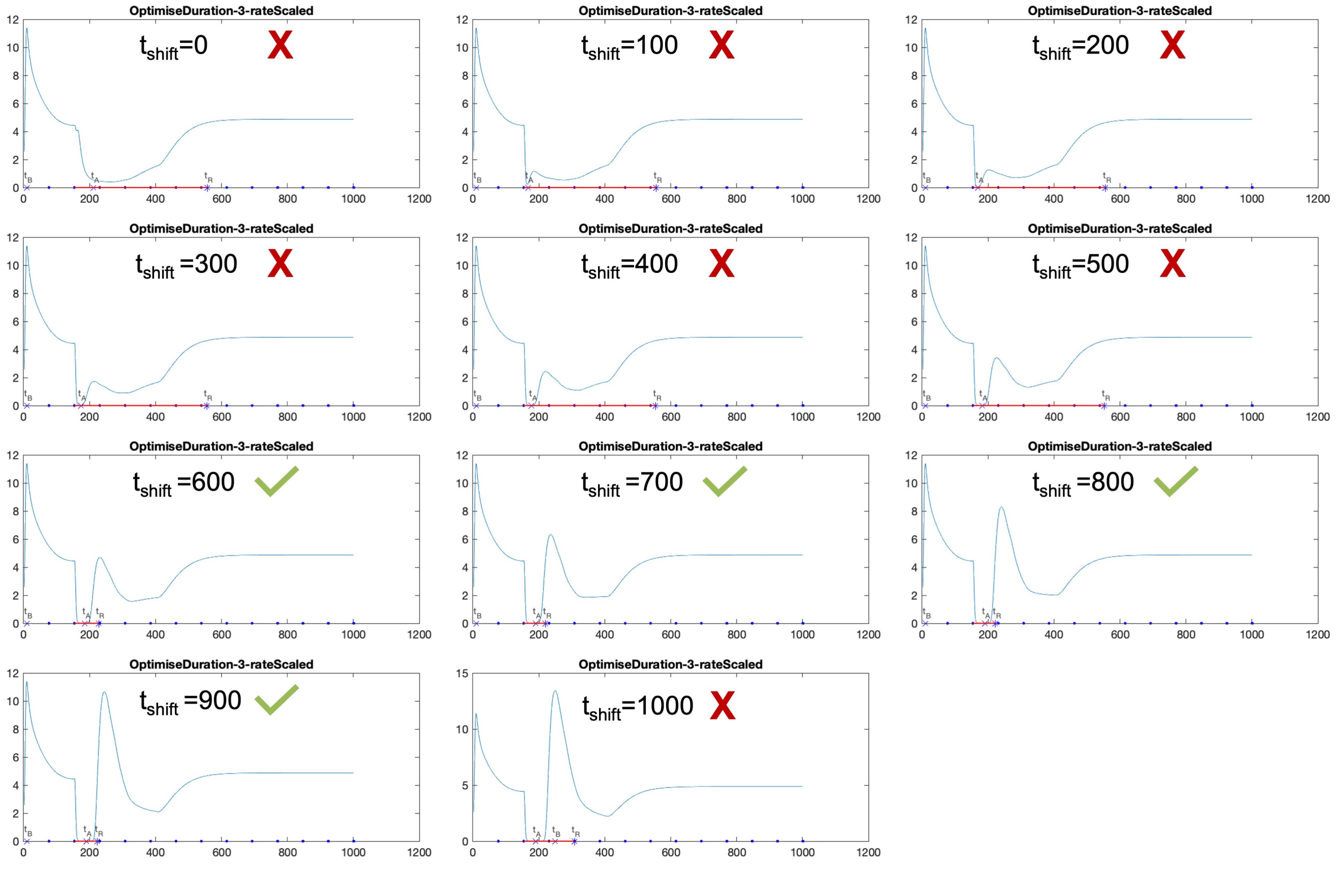

Figure S1: Examples of inferring symbol duration (t_s) values and selecting communication scenarios. Plots show the B_i/A_i ratios for communication scenarios for 11 t_{shift} values when α is 0.15. The plots with the red cross sign are for scenarios that are discarded since $t_{sInferred} > t_{sMax}$, while the plots with the green tick sign are for scenarios that are retained for further analysis. The time axis is scaled to the 0-1000 range. The data bits are 0010000000000, and $t_{sDefault}$ is 1500 s. Each time slot corresponds to 19.5 s (number_of_bits * $t_{sDefault}$ /1000).

Ranking selected communication scenarios using the MOL-eye performance metric

Ten communication scenarios were selected based on the maximum symbol duration criteria and then ranked. Figures S2 and S3 show the ranking of these scenarios from the lowest to the highest according to the MOL-eye performance evaluations. The diagrams on the left show A_i signals, while the diagrams on the right demonstrate the eye-opening patterns when A_i signals for bit-1 (blue lines) and bit-0 (red lines) symbols are overlayed.

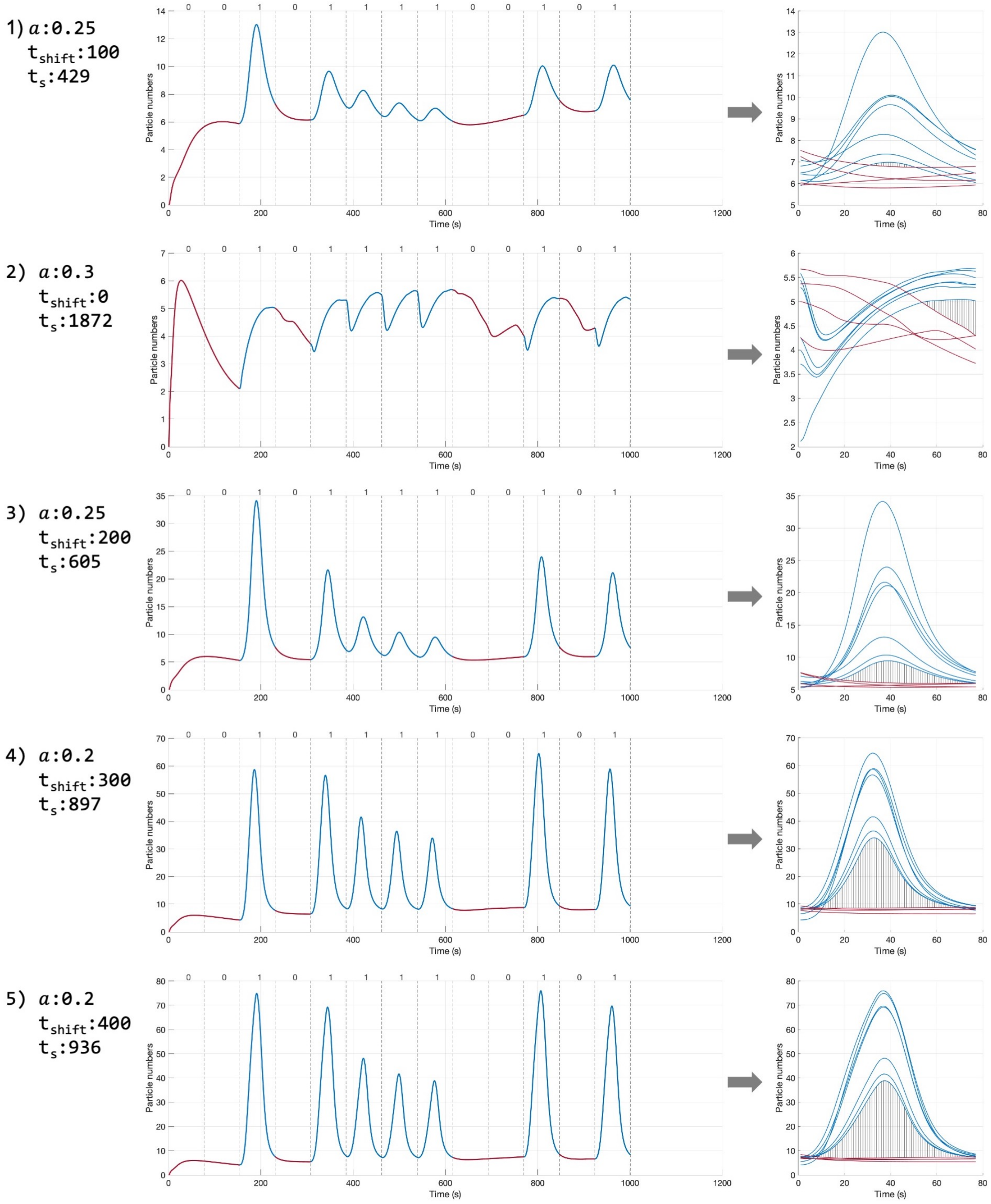

Figure S2: The first five communication scenarios with low scores.

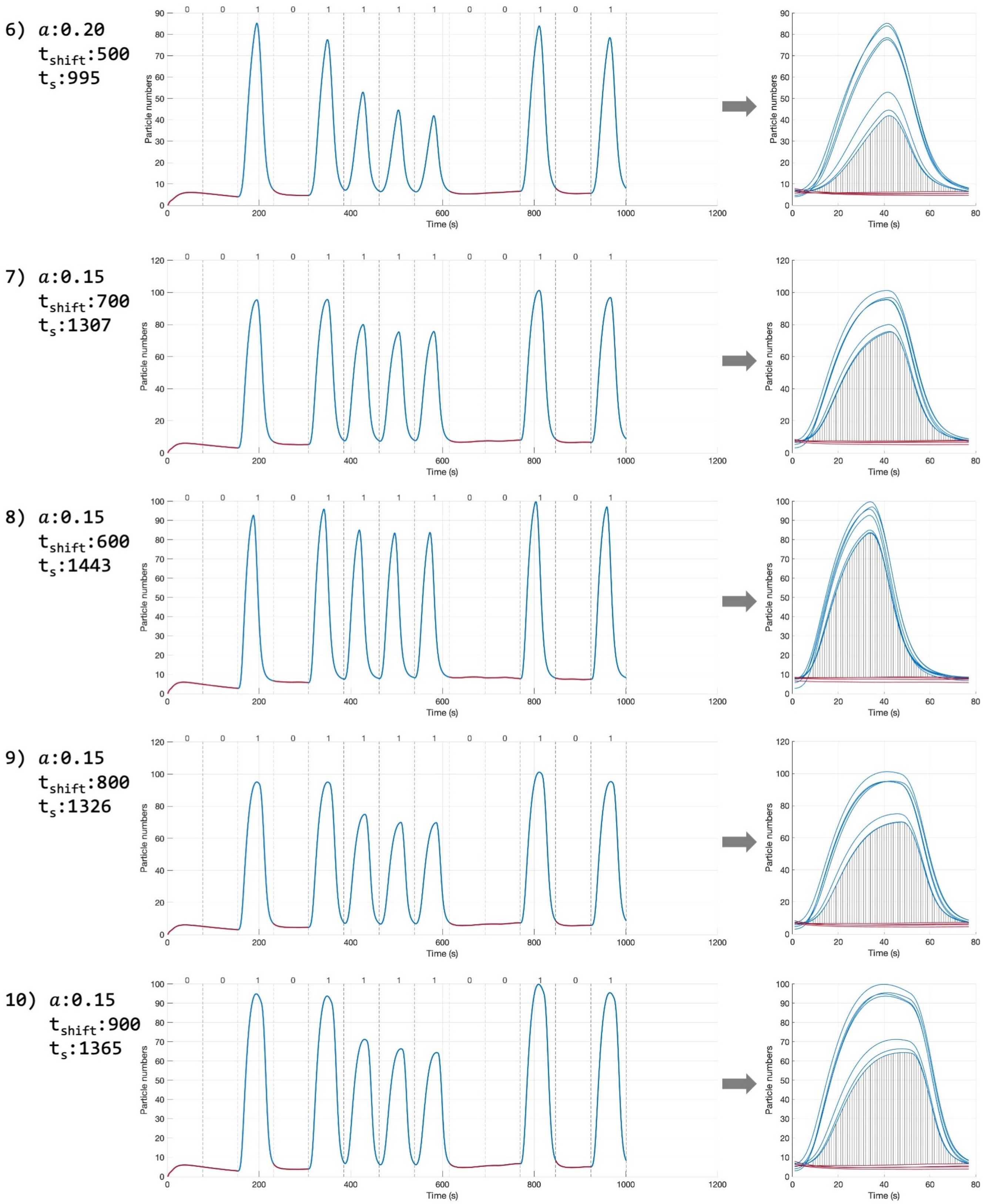

Figure S3: The last five communication scenarios with higher scores.

Dynamically creating models for communication scenarios

A hierarchical SBML model is created using the information about the order of genetic parts (Table S1) and the molecular interactions (Table S2). This model is then customised for communication scenarios using different α, t_{shift}, t_s and data bit values.

Table S1: The genetic circuit was designed as three devices using the SVPWrite language.

Device	SVPWrite construct
Sensor_Device	`pConstLacI:prom;rbs1:rbs;cdsLacI:cds;ter1:ter;pConstTetR:prom;rbs2:rbs;cdsTetR:cds;ter1:ter`
A_Device	`pLacI:prom;rbsLacI:rbs;cdsA:cds;ter1:ter`
B_Device	`pTetR:prom;rbsTetR:rbs;cdsB:cds;ter1:ter`

Table S2: Overview of the biological reactions represented in each model. Degradation reactions are not shown for simplicity. The arrows indicate the direction of the reactions between substrates and products. Modifiers are prefixed with the semicolon character.

	Reactions
Constitutive production of LacI and TetR	$pConstLacI \rightarrow mRNA_{LacI} \quad ;SigmaA$<br>$mRNA_{LacI} \rightarrow LacI$<br>$pConstTetR \rightarrow mRNA_{TetR} \quad ;SigmaA$<br>$mRNA_{TetR} \rightarrow TetR$
Production of B_i	$pTetR \rightarrow pTet + mRNA_B \quad ;SigmaA$<br>$mRNA_B \rightarrow B$<br>$pTetR + TetR_2 \leftrightarrow pTetR\_TetR_2$
Production of A_i	$pLacI \rightarrow pLacI + mRNA_A \quad ;SigmaA$<br>$mRNA_A \rightarrow A$<br>$pLacI + LacI_4 \leftrightarrow pLacI\_TetR_4$
Trans interactions	$TetR + TetR \leftrightarrow TetR_2$<br>$TetR_2 \rightarrow TetR_{aTc} \quad ;aTc$<br>$TetR_{aTc} \rightarrow TetR_2$<br>$LacI + LacI \leftrightarrow LacI_2$<br>$LacI_2 + LacI_2 \leftrightarrow LacI_4$<br>$LacI_4 \rightarrow LacI_{IPTG} \quad ;IPTG$<br>$LacI_{IPTG} \rightarrow LacI_4$<br>$A + B \leftrightarrow AB$

Table S3 shows the genetic parts' sequences and their sources. Sequences of parts and devices are also available as an SBOL version 2 file from the GitHub repository, described in the Data Availability section.

Table S3: Sequences of parts and their sources.

Part name	Sequences and sources
pLacI	Nielsen *et al.* [1], Supplementary Table 8 (LacI and TetR sensor module)<br>Nielsen *et al.* [1], Supplementary Table 9 (P_{LacI})<br>gcggcgcgccatcgaatggcgcaaaacctttcgcggtatggcatgatagcgcccgg
rbsLacI	Nielsen *et al.* [1], Supplementary Table 9 (LacI and TetR sensor module)<br>aagagagtcaattcagggtggtgaat
pTetR	Nielsen *et al.* [1], Supplementary Table 9 (P_{TetR})<br>tactccaccgttggctttttttccctatcagtgatagagattgacatccctatcagtgatagagataatgagcac
rbsTetR	Nielsen *et al.* [1], Supplementary Table 9 (LacI and TetR sensor module)<br>tccaggaggaaaaaa
ter1	Nielsen *et al.*, [1] Supplementary Table 9 (L3S2P21)<br>`https://parts.igem.org/Part:BBa_K2675031`<br>ctcggtaccaaattccagaaaagaggcctcccgaaagggggcctttttttcgttttggtcc
pConstLacI & pConstTetR	Nielsen *et al.* [1], Supplementary Table 9 (BBa_J23101)<br>`https://parts.igem.org/Part:BBa_J23101`<br>tttacagctagctcagtcctaggtattatgctagc
rbs1 & rbs2	Nielsen *et al.* [1], Supplementary Figure 34<br>`https://parts.igem.org/Part:BBa_B0064`<br>aaagaggggaaa
cdsA	Moon *et al.*, [2] Supplementary Table 4 (ExsD)<br>uniprot:P95429<br>atggagcaggaagacgataagcagtactcccgagaagcggtgttcgctggcaggcgggtatccgtggtgggctcggacgcccgctc<br>gcggggtcgggtgccgggttacgcatcgagcagtttgtatcgtgagtccggaatcatcagtgcgcggcaactggcgttgctgcagcgg<br>atgctgccgcgcctgcggctggagcaactgttccgctgcgagtggttgcagcagcgcctggcgcgcggcctggcgctggggcgcga<br>agaggtgcggcagattctcctctgcgcggcgcaggacgacgacggctggtgctccgaactgggcgaccgggtcaacctcgccgtgc<br>cgcagtcgatgatcgactgggtcctgctgccggtctatggctggtgggaaagcctgctcgaccaggcgatccccggctggcgcctgtc<br>gctggtggagctggagacccagtcccggcaactgcgagtcaagtccgaattctggtcccgcgtggccgagctggagccggagcagg<br>cccgcgaggaactggccagggtcgccaagtgccaggcgcgcacccaggaacaggtggccgaactggccggcaagctggagacg<br>gcttcggcactggcgaagagcgcctggccgaactggcagcggggcatggcgacgctgctcgccagcggcgggctggccggcttcg<br>agccgatccccgaggtcctcgaatgcctctggcaacctctctgccggctggacgacgacgtcggcgcggcggacgccgtccaggcct<br>ggctgcacgaacgcaacctgtgccaggcacaggatcacttctactggcagagctga
cdsB	Moon *et al.*, [2] Supplementary Table 4 (ExsA)<br>uniprot:P26993

	atgcaaggagccaaatctcttggccgaaagcagataacgtcttgtcattggaacattccaactttcgaatacagggtaaacaaggaagag<br>ggcgtatatgttctgctcgagggcgaactgaccgtccaggacatcgattccactttttgcctggcgcctggcgagttgcttttcgtccgccg<br>cggaagctatgtcgtaagtaccaagggaaaggacagccgaatactctggattccattatctgcccagtttctacaaggcttcgtccagcg<br>cttcggcgcgctgttgagtgaagtcgagcgttgcgacgagcccgtgccgggcatcatcgcgttcgctgccacgcctctgctggccggtt<br>gcgtcaaggggttgaaggaattgcttgtgcatgagcatccgccgatgctcgcctgcctgaagatcgaggagttgctgatgctcttcgcgt<br>tcagtccgcaggggccgctgctgatgtcggtcctgcggcaactgagcaaccggcatgtcgagcgtctgcagctattcatggagaagca<br>ctacctcaacgagtggaagctgtccgacttctcccgcgagttcggcatggggctgaccaccttcaaggagctgttcggcagtgtctatgg<br>ggtttcgccgcgcgcctggatcagcgagcggagaatcctctatgcccatcagttgctgctcaacagcgacatgagcatcgtcgacatcg<br>ccatggaggcgggcttttccagtcagtcctatttcacccagagctatcgccgccgtttcggctgcacgccgagccgctcgcggcaggg<br>gaaggacgaatgccgggctaaaaataactga
cdsLacI	Nielsen *et al.* [1], Supplementary Table 8 (LacI and TetR sensor module)<br>Nielsen *et al.* [1], Supplementary Table 9 (lacI)<br>atgaaaccagtaacgttatacgatgtcgcagagtatgccggtgtctcttatcagaccgtttcccgcgtggtgaaccaggccagccacgttt<br>ctgcgaaaacgcgggaaaaagtggaagcggcgatggcggagctgaattacattcccaaccgcgtggcacaacaactggcgggcaa<br>acagtcgttgctgattggcgttgccacctccagtctggccctgcacgcgccgtcgcaaattgtcgcggcgattaaatctcgcgccgatca<br>actgggtgccagcgtggtggtgtcgatggtagaacgaagcggcgtcgaagcctgtaaagcggcggtgcacaatcttctcgcgcaacg<br>cgtcagtgggctgatcattaactatccgctggatgaccaggatgccattgctgtggaagctgcctgcactaatgttccggcgttatttcttga<br>tgtctctgaccagacacccatcaacagtattattttctcccatgaggacggtacgcgactgggcgtggagcatctggtcgcattgggtcac<br>cagcaaatcgcgctgttagcgggcccattaagttctgtctcggcgcgtctgcgtctggctggctggcataaatatctcactcgcaatcaaa<br>ttcagccgatagcggaacgggaaggcgactggagtgccatgtccggttttcaacaaaccatgcaaatgctgaatgagggcatcgttccc<br>actgcgatgctggttgccaacgatcagatggcgctgggcgcaatgcgcgccattaccgagtccgggctgcgcgttggtgcggatatct<br>cggtagtgggatacgacgataccgaagatagctcatgttatatcccgccgttaaccaccatcaaacaggattttcgcctgctggggcaaa<br>ccagcgtggaccgcttgctgcaactctctcagggccaggcggtgaagggcaatcagctgttgccagtctcactggtgaaaagaaaaac<br>caccctggcgcccaatacgcaaaccgcctctccccgcgcgttggccgattcattaatgcagctggcacgacaggtttcccgactggaaa<br>gcgggcagtgataa
cdsTetR	Nielsen *et al.* [1], Supplementary Table 8 (LacI and TetR sensor module)<br>Nielsen *et al.* [1], Supplementary Table 9 (tetR)<br>atgtccagattagataaaagtaaagtgattaacagcgcattagagctgcttaatgaggtcggaatcgaaggtttaacaacccgtaaactcg<br>cccagaagctaggtgtagagcagcctacattgtattggcatgtaaaaaataagcgggctttgctcgacgccttagccattgagatgttaga<br>taggcaccatactcacttttgccctttagaaggggaaagctggcaagattttttacgtaataacgctaaaagttttagatgtgctttactaagt<br>catcgcgatggagcaaaagtacatttaggtacacggcctacagaaaaacagtatgaaactctcgaaaatcaattagcctttttatgccaac<br>aaggtttttcactagagaatgcattatatgcactcagcgctgtggggcattttactttaggttgcgtattggaagatcaagagcatcaagtcg<br>ctaaagaagaaagggaaacacctactactgatagtatgccgccattattacgacaagctatcgaattatttgatcaccaaggtgcagagc<br>cagccttcttattcggccttgaattgatcatatgcggattagaaaaacaacttaaatgtgaaagtgggtcctaataa

Running BioRxToolbox with different parameters

BioRxToolbox can be configured with different parameters such as the ratio of the B_e and A_e signals (α), the delay (t_{shift}) between these signals, the distance between the sender and the receiver, diffusion coefficients, and the receiver's size. For example, Figure S4 shows symbol durations for four distance values between the sender and receiver regarding ten α and 11 t_{shift} parameters.

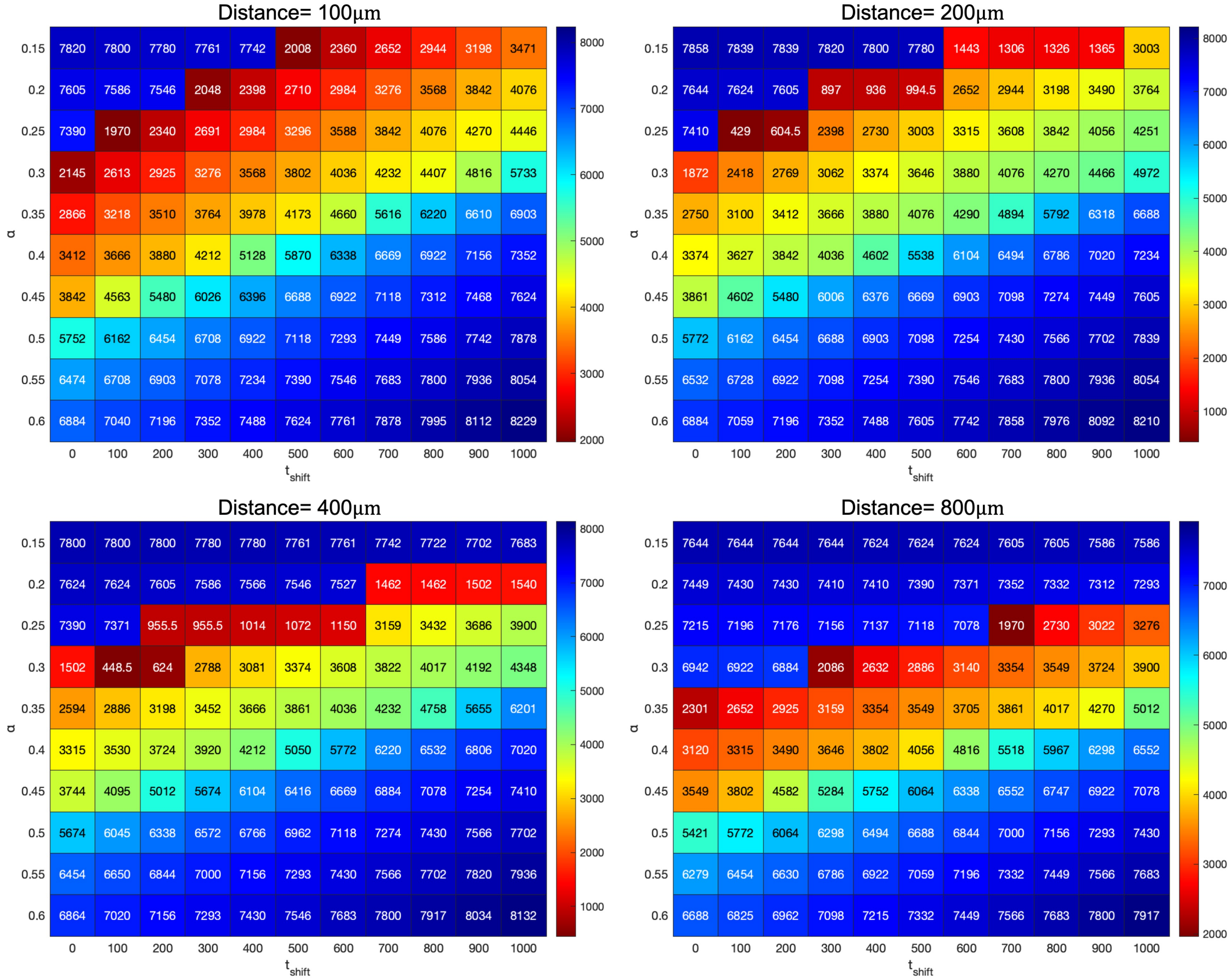

Figure S4: Symbol durations for four distance values between the sender and the receiver.

References

[1] Alec A. K. Nielsen, Bryan S. Der, Jonghyeon Shin, Prashant Vaidyanathan, Vanya Paralanov, Elizabeth A. Strychalski, David Ross, Douglas Densmore, and Christopher A. Voigt. Genetic circuit design automation. Science, 352(6281):aac7341, 2016.

[2] Tae Seok Moon, Chunbo Lou, Alvin Tamsir, Brynne C. Stanton, and Christopher A. Voigt. Genetic programs constructed from layered logic gates in single cells. Nature, 491(7423):249–253, 2012.
